# Morphological keys for identifying long-tailed gorals (*Naemorhedus caudatus*) and population composition in the Osaek Region of South Korea

**DOI:** 10.3897/BDJ.8.e58440

**Published:** 2020-11-17

**Authors:** Ki-Yoon Kim, Sang-Jin Lim, Jae-Yong Ahn, Ji-Hong Min, Yung-Chul Park

**Affiliations:** 1 Kangwon National University, Chuncheon, South Korea Kangwon National University Chuncheon South Korea; 2 Yanggu Goral Restoration Center, Yanggu, South Korea Yanggu Goral Restoration Center Yanggu South Korea; 3 Korea National Park Service, Wonju, South Korea Korea National Park Service Wonju South Korea

**Keywords:** Long-tailed goral, *Naemorhedus caudatus*, Seoraksan National Park, Osaek, morphological key, population composition, sex ratio

## Abstract

The objectives of this study were to select morphological keys for the identification of individual endangered long-tailed gorals through analysis of photographic data and to use these morphological keys to determine the number and population composition of gorals living in the Osaek Region of Seoraksan National Park. Amongst 8149 photos taken using 73 cameras in the Osaek Region, 2057 photos of faces and horns were analysed. The presence and absence of horns, shape of the horns, proportion of the ring to the length of the horn and facial colour pattern were selected as morphological keys to identify individual gorals. To verify the accuracy of the morphological keys for identifying gorals, a blind test was performed on gorals residing in the sanctuary of the Yanggu Goral Restoration Center. The test revealed that the population and age of gorals were discerned correctly by the morphological keys, but there was a 12.5% error in discriminating between sexes in gorals aged over 10 years. Fifty-six gorals were identified from 2057 pictures, based on the morphological keys and methods developed in this study. The population of 56 individuals consisted of 43 individuals aged over 2 years (subadult or adult) and 13 offspring aged less than 2 years, with a ratio of 3.3:1. Of the total 56 individuals, 45% were adults aged 10 years or older, 18% were adults aged 3–10 years, 7% were subadults aged 2–3 years, 23% were offspring aged less than 2 years and 7% were individuals aged 2 years or older, whose age and sex could not be confirmed. The sex ratio of males to females was 1.17:1, with a corrected sex ratio of 1:1 considering the 12.5% error rate for gorals aged over 10 years, amongst the 39 gorals aged over 2 years.

## Introduction

Small populations of wildlife can easily become extinct for a variety of reasons, including inbreeding, climate change, disease and invasive species (Shaffer 1981, Richman et al. 1988, Berger 1990, Schoener and Spiller 1992, Keeling 1997, Allendorf and Lundquist 2003, Hodgson et al. 2009, Wilder et al. 2020). The long-tailed goral, or Amur goral (*Naemorhedus caudatus*), is one of the wildlife species which has a small population and is threatened with extinction in South Korea. This species is an ungulate of the family Bovidae, which is found in a few forests or steep rocky areas in the mountains of the Korean Peninsula, north-eastern China and eastern Russia (Myslenkov and Voloshina 1989, Song et al. 2017, Jo et al. 2018). Recent studies using GPS collars (Cho et al. 2014, Cho et al. 2016) have shown that the goral is a sedentary animal with a relatively small home range of 0.58 to 1.46 km^2^. Unfavourable factors, such as the cultivation and fragmentation of habitats, have led to a decrease in the number of populations and this species is classified internationally as vulnerable (VU) on the IUCN (International Union for Conservation of Nature and Natural Resources) Red List (Bragina et al. 2020). In South Korea, it was designated as a natural monument No. 217 in 1997 and an endangered species in 1997 (Jo et al. 2018), with an estimated population of less than 1000 individuals (Song et al. 2017).

The Seoraksan National Park, along with Yanggu-Hwacheon, DMZ and Uljin-Samcheok, is one of the four largest goral habitats in Korea. Seoraksan National Park is located in the centre of the Baekdu-daegan, which is an elongated mountain range and watershed-crest-line that runs north to south through most of the length of the Korean Peninsula. Since the Seoraksan National Park has an excellent environment, like steep rocky areas, it maintains a population of more than 100 long-tailed goral individuals and plays an important role as a source population of gorals distributed in and around the Baekdu-daegan (Song et al. 2017).

The population size of long-tailed goral has only ever been estimated by visual inspection in Russia (Myslenkov and Voloshina 1989) and by morphometric (Cho et al. 2015, Kim et al. 2018) or genetic analysis (Choi et al. 2015, Jang et al. 2020) of faecal samples in South Korea. These methods need time-consuming efforts for the collection and analyses of faecal samples (Kim et al. 2018, Jang et al. 2020). Camera trapping provides accurate measurements of mammals since the repeated analysis of captured photos is possible and this is a more effective method than attempting to count by visual inspection in a field, because the rare individual is an endangered species or by morphometric analysis of faecal samples (Silveira et al. 2003, Kelly and Holub 2008, Ahumada et al. 2013). Camera-trapping has ever before been carried out for estimation of the long-tailed goral (Cho et al. 2015, Zaumyslova and Bondarchuk 2015, Park et al. 2018, Kim et al. 2020).

Our objectives were to select or improve the morphological keys that can identify individual gorals by analysing photographic data obtained through camera trapping, to test the accuracy of the morphological keys selected for goral identification and to estimate population size and composition of long-tailed gorals. In previous studies, long-tailed gorals were identified by morphological keys, such as horn shape, ring ratio on the horn, ring pattern on the horn and colour pattern on the face (Myslenkov and Voloshina 1989, Kwon et al. 2012). In this study, the morphological keys for identifying the gorals were newly selected or improved by analysing pictures taken with cameras installed in the Osaek. Finally, we identified the population size, sex ratio and age composition of gorals living in the Osaek Region using the selected morphological keys.

## Material and Methods

### Study area

**Figure 1.**
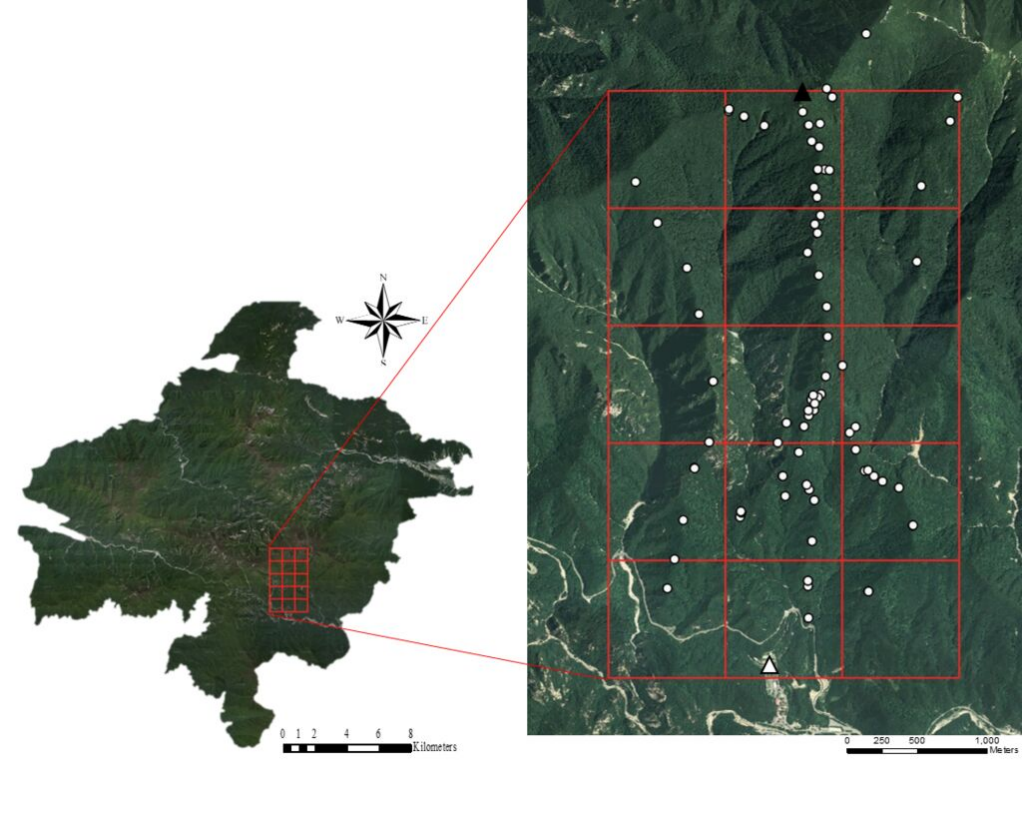
Seoraksan National Park (Left) and Study area with grids of Osaek Region in Seoraksan National Park. (▲: Kkeutchung Region △: Osaek village ○: Sensor camera location)

The Osaek Region of the Seoraksan National Park is an area that extends on both sides of the ridge that connects Kkeutcheong, located in the southwest of Daecheong-bong, the highest peak of the National Park, to Osaek village located in the south of Kkeutcheong. The study area was 9.6 km^2^, including the ridge where the cable car route is planned and was divided into 15 grids each with an area of 0.64 km^2^, with a width and length of 0.8 km (Fig. 1).

### Camera trapping

Long-tailed gorals live in forested areas surrounded by rocky cliffs and prefer rocks, slopes and cliffs in steep mountainous areas about 500-2000 m a.s.l. (Cho 2013, Cho et al. 2015, Jo et al. 2018). Sensor cameras (Spypoint’s Force-12 Canada and Moultrie's M-990i USA) were installed in places where goral excrement has been found or places suitable for rest through a line-transect survey in the Osaek Region. A total of 77 sensor cameras were installed at intervals of 350 ± 150 m in the area and a serial number was assigned to each camera (Fig. 1). Sensor cameras take pictures when sensing movements and these were programmed to take a picture every 24 h and obtain three images continuously with a delay time of 1 min between shots. The date, time and camera name are indicated on each photo. The picture quality was set to enhance resolution at 1080 p, which was the maximum quality. Sensor cameras were inspected every 2–3 months and the photographic data were downloaded during each inspection. The cameras operated from 9 November 2015 to 18 October 2016.

### Photo data for the selection of individual identification keys

Of the 8,149 goral images obtained by the cameras, 2057 were analysed to select new morphological keys or to improve the keys used in previous studies (Myslenkov and Voloshina 1989, Kwon et al. 2012). The selected 2057 images have a clear representation of morphological keys, excluding images with no faces and blurred photos taken on the move. The photos were divided into two groups, showing the front and side of the face of each goral. Photos of the front of the face were used for the primary analysis and photos of the side of the face were used for auxiliary analyses.

### Individual identification

Discriminating keys used to identify individuals, based on the photo data, were horn shape, proportion of the ring to the length of the horn and facial colour pattern. In addition, unique morphological features (e.g. a torn ear) were used as a secondary identification key when present. The pattern of ring at horns throughout the individual identification table (Kwon et al. 2012), used in the preceding study, was excluded from this individual identification index because it can be distorted due to the viewing angle of the sensor camera or intensity of light. To reduce the subjective elements of facial colour patterns presented in the preceding study (Kwon et al. 2012), two-colour quantification, which is typically expressed, were applied.

**Figure 2.**
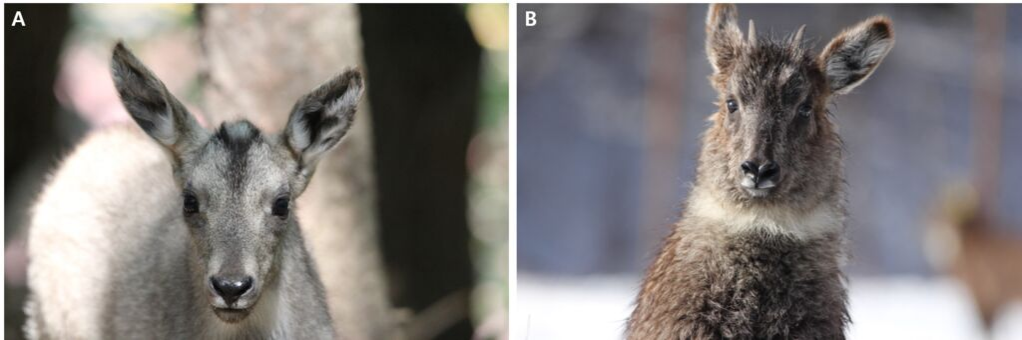
Goral offspring aged less than 2 years can be classified into two groups: (A) individuals born during the breeding period of the year in which the offspring were photographed and (B) those born during the breeding period of the year preceding that in which the goral offspring were photographed. Thus, the offspring in the first group were aged less than 0.5 years, with horns that had not yet appeared, while those in the second group were aged 0.5–1.5-years, with horns protruding above the fur in the centre of the forehead (photos of offspring were taken at the Yanggu Goral Restoration Center).

Long-tailed goral adults and offspring were classified by their body size, horn maturity and appearance of a discernible ring. While subadults (2–3-years old) and adults (more than 3-years old) have keratinised horns and discernible horn rings, the young (less than 2-years old) have an invisible horn or small horns that are undergoing keratinisation (Fig. 2). In addition, in offspring, the hairs in the centre of the forehead of the face are longer than those in the other parts of the face and white hairs and black hairs are mixed (Fig. 2). Using these features, the adult (A) and offspring (Y) were identified and assigned identification codes based on the photographic data (Table 1).

**Figure 3.**
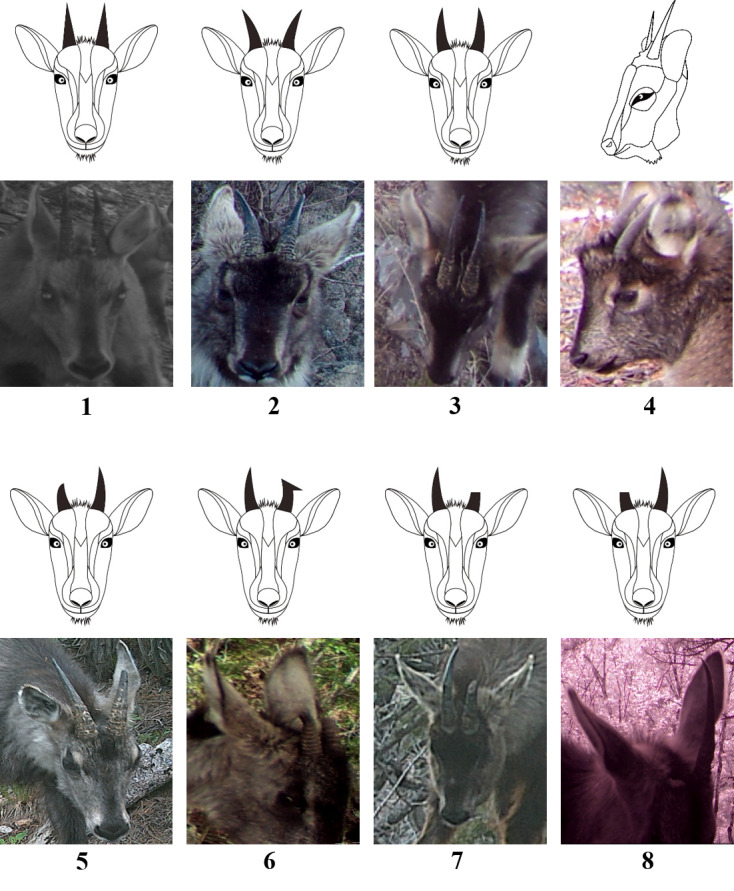
Photos and drawings of adult goral horns. Codes 1–8 beneath the photos correspond to the codes in Table 2 (both horns are the same shape as codes 1 to 4, but a different shape to codes 5–8. Detailed descriptions of each code are provided in Table 2).

**Figure 4.**
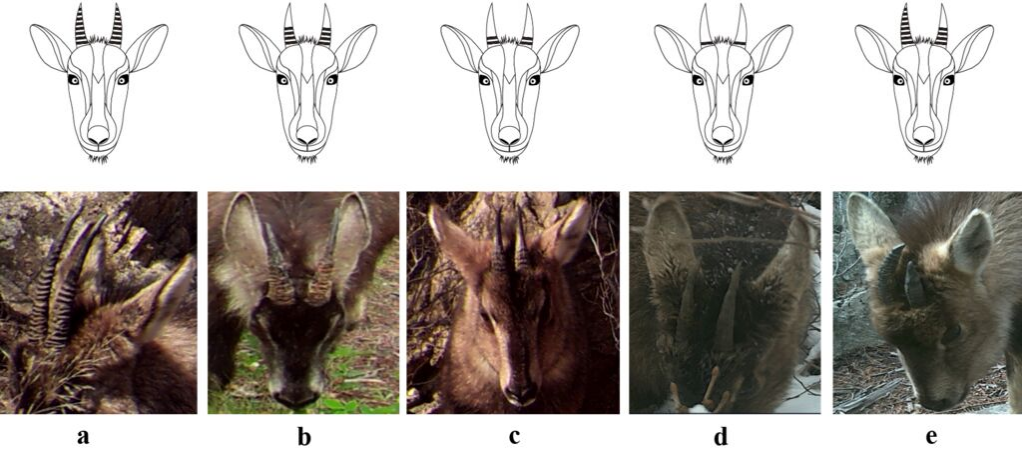
Photos and drawings of the rings on adult goral horns. Codes a–e beneath the photos correspond to those in Table 3 (both horn rings are the same shape and size as codes a–d, but different from code e. Detailed descriptions for each code are provided in Table 3).

**Figure 5.**
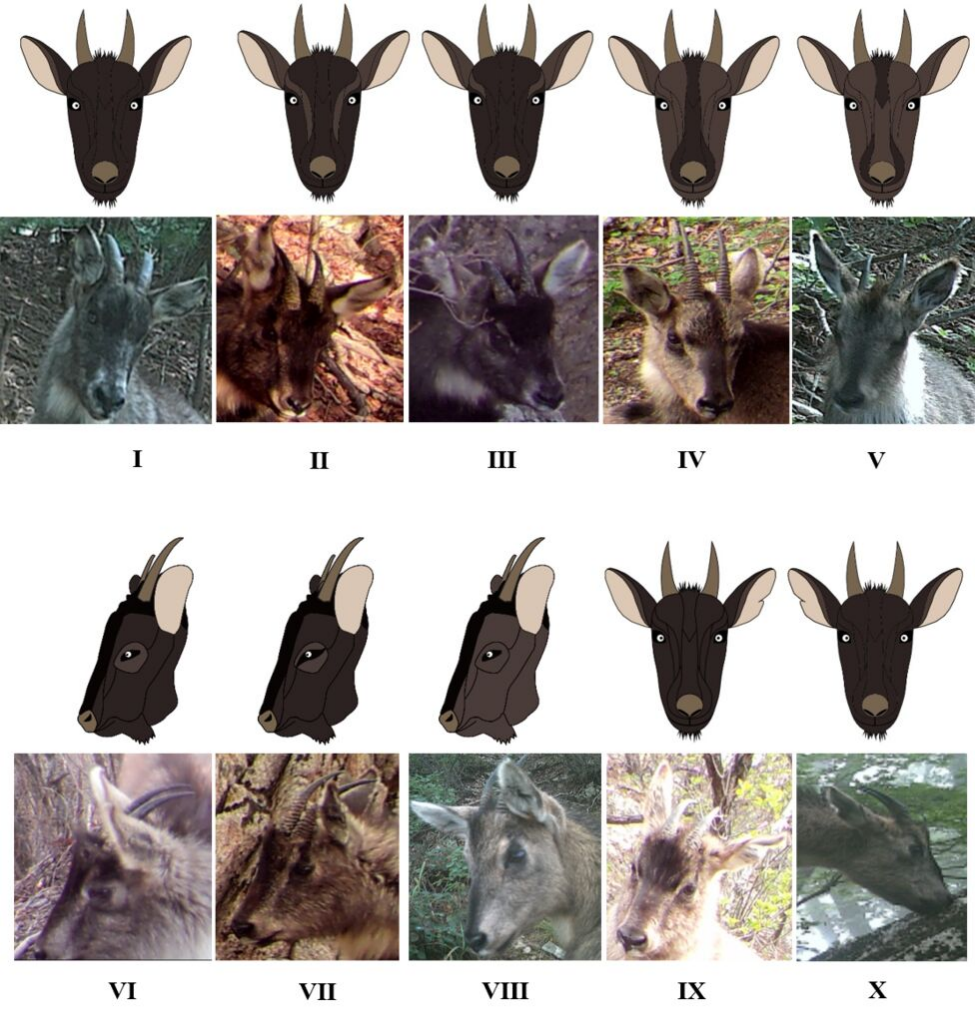
Photos and drawings of the facial colour pattern of gorals. Codes I–X beneath the photos correspond to those in Table 4.

Horns of goral infants born in the summer were not visible in the year of their birth (Fig. 2A) and appeared above the fur in the centre of the forehead after the end of December of the year following their birth (Fig. 2B). Therefore, it was possible to determine the year of birth, based on the presence or absence of horns above the hair of infants. Using this feature, goral infants could be divided into a group in which the horns were hidden by their fur on the forehead (I) (less than 1-year-old) (Fig. 2A) and a group in which the horns were protruding above the hair (V) (1–2 years old) (Fig. 2B). The horn shape could be divided into two types, based on their shape: regular shape and irregular shape. Both regular and irregular shapes were further subdivided into four types. Therefore, horn shape could be subdivided into eight types, which were assigned codes 1–8 (Fig. 3, Table 2). The proportion of rings on both horns was divided into a group where the ring proportion was the same for both horns and a group where the ring proportion differed between horns. When the ring proportion was the same in both horns, this group was subdivided into four types (less than 20%, 21–50%, 51–70% and 71% or more). Therefore, the ring proportion was subdivided into five groups and assigned codes a–e (Fig. 4, Table 3). The horn ring pattern used in a previous study (Kwon et al. 2012) was excluded from the analysis because it may have been distorted by the viewing angle of the camera and/or the light intensity. The facial colour pattern was divided, based on the percentage of black across the entire face (I–X) (Fig. 5, Table 4).

### Age identification

The age of individuals, excluding those under 2 years, was assessed using the proportion of horn rings, starting at the base of the horns according to the method described by Myslenkov and Voloshina (1989). For offspring aged less than 2-years, a lack of visible horns indicated that they were born during the breeding period of the year (less than 0.5 years old). If small horns were visible on the hairs of the forehead, but the rings could not be identified, this indicated they were born in the previous year (0.5–2 years old). In subadults, the rings of the horns were identified and the proportion of the ring to horn length was less than 20% (2–3 years old). Adults were divided into three groups according to the proportion of the horn covered by the ring. The ring proportion was 21–50% in adults aged 3–10 years and more than 50% in adults aged 10 years or older (Table 3). Since goral horns do not regrow once they are broken, age was measured, based on the ring proportion of the remaining intact horn. If the proportion of the ring on both horns could not be determined, the age was classified as unidentified.

### Individual code generation

Each photo was assigned an individual goral code by sequentially arranging identification codes (Table 5). For the generation of individual goral codes, the photo data were first classified into adult (A) and offspring (less than 2-years of age) (Y). In the case of adult photos, the codes were sequentially identified and arranged in the order of the horn shape (1–8), the proportion of the ring to horn length (a–e) and face colour pattern (I–X). Therefore, an individual four code, in which each digit was sequentially arranged, was assigned to each goral photo. In each individual four code, ‘*’ denotes where identification key information is missing.

For offspring (Y) aged less than 2 years, the photos were first classified into cases where the horns were visible (V) and the horns were not visible (I). Then, the facial colour pattern code (I–X) was assigned to the photo data. In offspring aged less than 2 years, * was used to denote the third position of the four-digit code (Table 5) because the shape and ring proportion of the horn could not be determined. Photos with an individual goral code were sorted using Excel. Photos with the same four-digit code and those with one difference in the code, were reviewed while considering the presence of other morphological distinctions not included in the identification codes. Although they had the same individual code, offspring aged less than 2 years were sorted as different individuals if their mothers were different.

### Sex identification

Facial photos of six females with offspring were analysed to distinguish gender. In females, 80% or less of the entire area of the face was black and the black central pattern from the forehead to the tip of the nose was clearly observed. In contrast, in males, black occupied more than 80% of the facial area and the black central pattern was not clearly distinguished. The difference in colour patterns between males and females was consistent with that used by Myslenkov and Voloshina (1989) for the gender classification of long-tailed gorals in Russia. Thus, adult females and males could be distinguished by the proportion of black on the face and the black central pattern from the forehead to the tip of the nose.

### Verification of the accuracy of individual identification

A blind test was performed to verify the accuracy of the morphological keys and sorting method used in this study to identify goral individuals using photographic data. Eight cameras were installed within 0.98 km^2^ in forests of the goral sanctuary of the Yanggu Goral Restoration Center, Gangwon Province. Both numbers of cameras and locations were determined to take photos of all individuals in the goral sanctuary considering their home range of about 0.58 to 1.46 km^2^. In addition, to obtain images of goral individuals with recognisable morphological keys, the period of camera-trapping was set for more than a week. The gorals in that area were photographed for 2 weeks from 10-23 September 2016.

Without any information on the population of gorals living in the sanctuary forests, the photo data were analysed by applying the identification keys and the sorting method employed in this study. Analyses, based on photographic data, were sent to the Yanggu Goral Restoration Center to verify the accuracy of the analysis and determine the error rate by comparing the results with information on gorals currently residing in the sanctuary.

## Results and Discussion

Overall, 3,330 photos of gorals were taken by the eight cameras installed in the goral sanctuary of the Yanggu Goral Restoration Center. Of those, 624 facial photos were used for the identification of goral individuals.

All 18 goral individuals, including three offspring aged less than 1 year, were identified by analysing the photographic data according to the described identification keys and sorting method (Table 6). Excluding the three offspring aged less than 1 year, 13 of the 15 individuals were classified as adults aged 3 years or older and two individuals had broken horns, meaning their age could not be confirmed. Excluding the offspring and two individuals of unconfirmed age, 11 males and two females were identified amongst 13 adults older than 3 years and seven males and one female amongst eight adults aged over 10 years. In the 15 individuals (including two of unconfirmed age), 11 males and four females were identified. The sex of the three offspring could not be determined (Table 6, Table 7).

The list of gorals living in the goral sanctuary provided by the Yanggu Goral Restoration Center included 18 individuals, which was consistent with the number of gorals analysed in this study. This indicated that the method of goral identification used in the study was highly accurate (Table 6). The two individuals whose age could not be confirmed due to broken horns were identified as female offspring aged 1–2 years, based on the list.

Based on the photographic analysis, seven males (87.5%) and one female (12.5%) were identified amongst the eight adults aged 10 years or older; however, in the list, there were six males (75%) and two females (25%) amongst the eight adults (Table 7). Therefore, compared with the actual data, there was a 12.5% error in the sex identification of adults aged 10 years or older, based on the analysis of photo data. For similar studies, photo identification of Indonesian rhinos estimated the mean population size with a standard error of 19.07% (Hariyade et al. 2011). In snow leopards, a population estimation using photographs showed an error of 12.5% (Johansson et al. 2020).

For estimation of population size and composition of long-tailed gorals in Osaek, a total of 8149 goral photos were obtained by 73 out of 77 cameras (four broken cameras were excluded) installed in the Osaek Region of Seoraksan National Park. From these, 2057 facial photos were used to identify gorals. Fifty-six gorals were identified from the photos, based on the morphological keys and methods developed in this study. Individuals, aged less than 2 years, were difficult to identify by the morphological keys; however, they were identified, based on their mothers.

**Figure 6.**
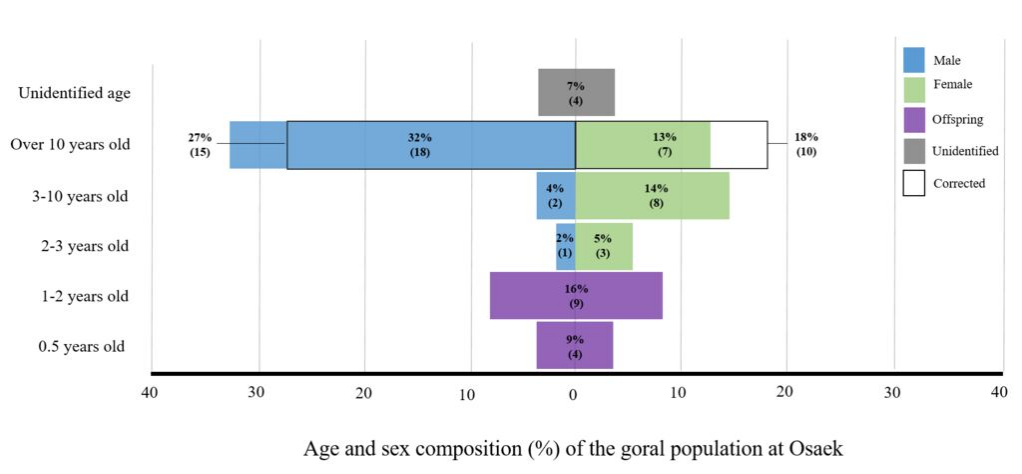
Sex and age composition of 56 gorals living in the Osaek Region.

The population consisted of 43 individuals aged over 2 years (subadult or adult) and 13 offspring aged less than 2 years, at a ratio of 3.3:1 (Fig. 6). Neither age nor sex could be identified in 4 of 43 gorals that were in the subadult or adult groups. Of the 56 total individuals, 45% were adults aged 10 years or older, 18% were adults aged 3–10 years, 7% were subadults aged 2–3 years, 23% were offspring aged less than 2 years and 7% were individuals aged 2 years or older, whose exact age and sex could not be identified (Fig. 6).

Amongst the 10 adults aged 3–10 years and four subadults aged 2–3 years, there was a higher percentage of females than males. Conversely, amongst the 25 adults aged 10 years and over, there was a higher percentage of males than females (Fig. 6). In the verification of accuracy (Table 7), the error rate at which females could be judged as male was 12.5% in adults aged over 10 years. Thus, three individuals, representing 12.5% of the 25 adults aged over 10 years, were removed from the number of males and added to the number of females. Consequently, the corrected number of males and females was 15 and 10 in this group, respectively (Fig. 6).

The sex ratio of males to females was 1.17:1, with a corrected ratio of 1:1, amongst 39 gorals aged over 2 years, excluding offspring and the four unidentified individuals. The sex ratio of males to females was 0.3:1 in four subadults, 0.3:1 in 10 adults aged 3–10 years and 2.6:1 in 25 adults aged over 10 years, Notably, in adults aged over 10, the corrected sex ratio was 1.5:1.

According to a previous study by 10 camera trappings in the Sikhote-Alin Reserve in Russia in 2015 (Zaumyslova and Bondarchuk 2015), adult females (28.8%) have a higher proportion than males (20%) in the number of long-tailed gorals. In surveys of the goral population performed in Abrek Urochische in Russia in 1975-1976, the sex ratio of male and female was 1:2 in 1975 and 1:1.6 in 1976 (Myslenkov and Voloshina 1989). Female-biased sex ratios were also observed in a subspecies of long-tailed goral, Himalayan grey goral (Abbas et al. 2012). In the study, analysis of sex ratio in relation to ages indicates that the Osaek population under 10 years old showed a female-biased sex ratio which is similar to that of the long-tailed gorals in Sikhote-Alin and Abrek in Russia. In adults over 10 years, however, a male-biased sex ratio of 1.5:1 was observed. Disturbance of goral habitats within Seoraksan National Park has increased gradually by visitors and mountain trails. Thus, male-biased sex ratio in adults over 10 years might be the result of the flow of male adults into Osaek Region which has greater conservation measures in comparison with other regions of Seoraksan National Park.

In conclusion, individual differences in long-tailed goral horns and facial colours employed in the study could be used as useful natural identification markers for photo identification in camera trapping. Population size and composition estimated by camera trapping indicate that long-tailed gorals of high density with various age classes including yearlings inhabit the Osaek Region. Thus, the Region should be well conserved as important habitats for the endangered gorals.

## Figures and Tables

**Table 1.** Morphological keys used for the classification of subadult or adult (2 years or older) long-tailed gorals and offspring (aged less than 2 years).

Age class	Morphological key	Code
Adult (A) and subadult	Keratinised horns with discernible rings are visible	A (A)
Young (Y)	Small horns that are undergoing keratinisation are visible, but the horn ring is not discernible	V (Y–V)
	The horns have not yet appeared	I (Y–I)

**Table 2.** Morphological keys of horns used for the identification of individuals gorals (subadult or adult).

Morphological key	Classification of morphological key	Detailed classification of morphological key	Code^*^
Shape of horns above the forehead	Horns above the forehead are identical in shape	Viewed from the front, both horns extend parallel upwards (| |)	1
		Viewed from the front, the ends of each horn bend outwards (\ /)	2
		Viewed from the front, the ends of each horn bend inwards (/ \)	3
		Viewed from the side, the horns do not bend backwards (//)	4
	Horns above the forehead are not identical in shape	Viewed from the front, the horns bend at different angles	5
		Viewed from the front, horn shape differs	6
		The left horn is broken.	7
		The right horn is broken.	8

- ^*^Photos corresponding to each code are shown in Fig. 3.

**Table 3.** Morphological keys of horn rings for the identification and age classification of individual gorals aged 2 years and older.

Morphological key	Classification of morphological key	Detailed classification of morphological key (proportion of the horn included in the ring)	Code	Age class	
Shape and area of horn rings	Rings on the both horns are the same in area and shape	71% or more	a	10 years or older	Adult
		51–70%	b	10 years or older	
		21–50%	c	3–10 years	
		20% or less	d	2–3 years	Subadult
	Rings on the both horns are different in area and shape	Irregular	e	Unidentified	

- ^*^Photos corresponding to each code are shown in Fig. 4.

**Table 4.** Individual identification and sex classification, based on the adult facial colour pattern.

Fig. 5 Morphological key	Classification of morphological key		Detailed classification of morphological key	Code	Sex
Facial colour pattern	Front view (from the forehead to the tip of nose)	80% or more of the face is black	Whole face is black	Ⅰ	M
			White stripes on both sides, from the left and right sides of the forehead to the nose	Ⅱ	
			White stripes around both eyes and the centre of the forehead	Ⅲ	
		Less than 80% of the face is black	Black stripes from the centre of the forehead to the tip of the nose	Ⅳ	F
			Short black vertical stripes in the middle of the forehead and black stripes around the tip of the nose	Ⅴ	
	Side view (from the eyes to the base of the horn)	More than 70% of the face is black	White circular pattern surrounding the eyes	Ⅵ	M
			White circular pattern surrounding the eyes is split by a black line	Ⅶ	
		Less than 70% of the face is black	Black circular and horizontal line around the eyes	Ⅷ	F
Other morphological feature			Torn left ear	Ⅸ	
			Torn right ear	Ⅹ	

- ^*^Photos corresponding to each code are shown in Fig. 5.

**Table 5.** Step-by-step process of assigning an identification code to each goral.

Step	Step 1			Step 2	Step 3	Step 4	Assigned individual code
Morphological key	Adult or subadult/offspring			Horn shape	Proportion of horn ring	Facial colour pattern	
Code	adult or subadult	A		(1–8)	(a–e)	(Ⅰ–Ⅹ)	A-(1–8)-(a–e)-(Ⅰ–Ⅹ)
	Offspring	Y	Invisible small horn (I)	Not applicable (*)			Y-(I or V)-*-(Ⅰ–Ⅹ)
			Visible small horns (V)				

**Table 6.** Goral code, sex and age obtained by analysing photos taken by cameras installed in the sanctuary of Yanggu Goral Restoration Center and information on gorals residing in the area.

Individual information on gorals residing in the sanctuary			Individual information obtained through the analysis of photo data using morphological keys		
Individual No.	Sex	Age	Individual code	Sex	Age
01	M	Adult older than 10 years (＜)	A-2-a-Ⅰ	M	Adult aged 10 years or older (≤)
02	M	Adult aged 10 years (=)	A-3-b-Ⅲ	M	Adult aged 10 years or older (≤)
03	M	Adult older than 10 years	A-7-e-Ⅱ	M	Adult aged 10 years or older
04	F	Offspring 1–2-years old (born April 18, 2015)	A-7,8-*-Ⅳ^+^	F	Unidentified
05	-	Offspring less than 1-year old (born April 2, 2016)	Y-I-*-Ⅱ	-	Offspring less than 1-year old
06	M	Adult aged 3–10 years old	A-1-c-Ⅲ	M	Adult aged 3–10 years
07	F	Offspring aged 1–2 years (born 17 Jun 2015)	A-7,8-*-Ⅳ^+^	F	Unidentified
08	F	Adult aged 3–10 years	A-1-c-Ⅳ	F	Adult aged 3–10 years
09	^F^	Adult older than 10 years	A-2-b-Ⅲ	M	Adult aged 10 years or older
10	-	Offspring less than 1-year old (born 17 May 2016)	Y-I-*-Ⅳ	-	Offspring less than 1-year old
11	M	Adult older than 10 years	A-7-b-Ⅲ	M	Adult aged 10 years or older
12	M	Adult aged 3–10 years	A-8-c-Ⅱ	M	Adult aged 3–10 years
13	M	Adult aged 3–10 years	A-2-c-Ⅲ	M	Adult aged 3–10 years
14	M	Adult aged 3–10 years	A-1-c-Ⅲ	M	Adult aged 3–10 years
15	M	Adult older than 10 years	A-7-b-Ⅱ	M	Adult aged 10 years or older
16	F	Adult older than 10 years	A-2-b-Ⅳ	F	Adult aged 10 years or older
17	M	Adult aged 10 years	A-3-b-Ⅲ	M	Adult aged 10 years or older
18	-	Offspring less than 1-year old (born 26 May 2016)	Y-I-*-Ⅴ	-	Offspring less than 1-year old

- ^+^ If both horns are broken, codes 7 (the left horn is broken) and 8 (the right horn is broken) are given.

- * indicates an unidentified or missing code in the four-digit individual code.

**Table 7.** Comparison between goral information obtained by analysing photos taken by cameras at the goral sanctuary and information on gorals residing in the sanctuary of the Yanggu Goral Restoration Center.

Sex	Age									
	Adult				Subadult		Offspring		Unidentified	
	10-years old or older		3–10-years old		2–3-years old		Less than 1-year old			
	MKSM	KGRC	MKSM	KGRC	MKSM	KGRC	MKSM	KGRC	MKSM	KGRC
M	7	6	4	4	0	0	3	3	0	0
F	1	2	1	1	0	2			2	0
Total	8	8	5	5	0	2	3	3	2	0

- MKSM: Goral information obtained by analysing photos taken with eight cameras installed in the goral sanctuary of the Yanggu Goral Restoration Center using the morphological keys and sorting method developed in this study.

- KGRC: Information on gorals residing in the goral sanctuary provided by the Yanggu Goral Restoration Center.
